# The Impact of the COVID-19 Pandemic on the Professional Autonomy of Anesthesiological Nurses and Trust in the Therapeutic Team of Intensive Therapy Units—Polish Multicentre Study

**DOI:** 10.3390/ijerph191912755

**Published:** 2022-10-05

**Authors:** Hanna Krukowska-Sitek, Sabina Krupa, Iga Grad

**Affiliations:** 1 Institute of Health Sciences, Medical College of Rzeszow University, Warzywna St. 1, 35-310 Rzeszow, Poland; 2 Faculty of Health Sciences, University of Opole, Katowicka St. 68, 45-060 Opole, Poland

**Keywords:** pandemic, COVID-19, professional autonomy, trust, therapeutic team, ICU

## Abstract

Introduction: The COVID-19 pandemic as well as the rate of spread of this particular pathogen around the world have caused the number of patients requiring medical attention and intensive care to exceed the capacity of even the best organized health care systems. This resulted in the need to hire employees who had not previously worked in intensive care units. Experience and knowledge have become particularly important in the context of mutual trust in the ICU team. At the same time, it could affect the level of professional autonomy of nurses, understood as the freedom to perform work based on knowledge, skills and competence without the need to submit to other medical professions. The pandemic status has required that nurses are always involved in their work by participating in training. Faced with the dangers of COVID-19, there is no doubt that by the end of the pandemic, both nursing and healthcare will be better equipped to face future challenges. Methods: The study lasted from July to September 2021. The data collection procedure started with the consent of the heads of the institutions where the data was collected. The study was conducted using the Dempster Practice Behavior Scale (DPBS), which examines work autonomy. The survey using the proprietary questionnaire was conducted among 225 nurses working in eleven ICUs in five voivodeships in Poland. Results: The autonomy of nurses during the COVID-19 pandemic was assessed at a high level. Younger respondents rated autonomy as being higher. Almost half of the respondents assessed the level of professional independence as high, including 52% of nurses, and significantly less, including 34% of doctors. A group of 47% of respondents assessed that trust had decreased and 28% said that trust had improved slightly. Conclusions: Professional independence allows you to perform work independently on others, taking responsibility for decisions and actions. The COVID-19 pandemic, through the influx of new staff members into treatment teams, had an impact on both nursing autonomy and the level of trust in a team, as shown in this study.

## 1. Introduction

The end of the second decade of the 21st century brought fear and terror to the world over the emergence of a new deadly pathogen. The first symptoms of the disease, indicating an acute respiratory infection, were observed in December 2019 in Wuhan, Hubei Province, China [1], and just 4 months later, the World Health Organization classified the disease as the fifth pandemic affecting humanity since the first Spanish flu pandemic after 1918, and the first documented pandemic caused by pathogens from the coronavirus group [2]. During the COVID-19 pandemic, the COVID (+) units significantly increased the autonomy of nurses, as the number of staff working with patients was limited. For hospital decision makers, it was important to enable nurses to participate in decision-making and to develop nursing through shared leadership so as to increase the maintenance of a skilled workforce [3]. Despite the existence of inquiries into the qualifications of nurses, it is believed that nurses ‘autonomy contributes to the efficiency of nurses’ work, as they show their full potential and increase their professional autonomy [4]. It should be remembered that professional autonomy of a nurse exists, when nurse having moral and intellectual independence, uses the ability to follow her or his own experience, makes independent decisions about her or his own practice, thanks to which she or he is able to make informed decisions among the available options. When an employee has a high level of autonomy, nurse can give actions value and social recognition, which also positively affects nurses job satisfaction [5]. The pandemic status has required that nurses are always involved in their work by participating in training. Faced with the dangers of the virus in the world, there is no doubt that by the end of the pandemic, both nursing and healthcare will be better equipped to face future challenges [6].

## 2. Materials and Methods

### 2.1. Design

The cross-sectional study was conducted. The project was implemented in the Intensive Care Units in Poland.

### 2.2. Setting and Procedure

This article was written in accordance with the STROBE (Strengthening the Reporting of Observational Studies in Epidemiology) guidelines to improve the quality of reporting this study [7]. The study lasted from July to September 2021. The data collection procedure was started with the consent of the heads of the institutions where the data was collected. The next step was the Bioethics Committee’s approval. The study was positively assessed by the Bioethics Committee of the University of Rzeszów (No. 10/05/2021). The participants of the study received information about the purpose and method of conducting the survey. They were also informed about the possibility of withdrawing from participation in the study at any time during its duration. The respondents were informed about the method of giving the informed consent to participate in the study by correctly filling in and returning the questionnaire to the researcher. The objectives of the study were validation through the Dempster Practice Behavior Scale into Polish and evaluation the impact of the COVID-19 Pandemic to the professional autonomy of anesthesiological nurses and trust in the therapeutic team of intensive therapy units.

The main research question was: did the COVID-19 pandemic affect nursing autonomy and trust in the ICU team?

### 2.3. Participants

The survey using the proprietary questionnaire, containing responses presented by introducing the 4-point Lickert scale, was conducted among 225 nurses working in 11 ICUs in 5 voivodeships in Poland: Łódzkie, Małopolskie, Podkarpackie, Świętokrzyskie and Warmińsko-Mazurskie, and in a group of 47 anaesthesiologists working in 8 ICUs in 5 voivodeships: Łódzkie, Małopolskie, Podkarpackie, Śląskie and Świętokrzyskie, in the period from July to September 2021. These 5 voivodeships were selected through authors professional work—the authors had the opportunity to hand out the questionnaires in person by staying in these hospitals.

### 2.4. Data Sources/Measurement

#### Dempster Practice Behavior Scale (DPBS)

The DPBS scale is a tool consisting of 30 items. It was created on the basis of an analysis of empirical and theoretical literature in the field of many disciplines, in accordance with the principles of creating research tools, i.e., the use of validation stages, a panel of experts and psychometric tests. The adopted forms are not specific to the field of nursing, which allows the use of the tool to test the level of autonomy in other disciplines. The scale includes both positive and negative wording. All phrases are preceded by a statement referring to the examined person “at the workplace...”. The respondents express his or her attitude to the statement on a 5-point Likert scale, on which subsequent statements were assigned to an appropriate point value in the range 1–5, where 1 means complete disagreement with the statement to which the answer relates, and the value 5 means complete agreement with a given statement. In several statements, reverse scoring was used, which means that the value 1 should be assigned with 5 points and the value 5 should be assigned with 1 point. Based on the given answers, 30–150 points can be awarded. In the case of the overall score, the higher the score, the greater the respondent’s range of autonomy [8].

### 2.5. Statistical Analysis

Data were collected for the DPBS, Jefferson, and NCCCS tests. For these tests data, a reliability analysis was performed to assess the reliability of the analyzed scales by calculating the Cronbach’s alpha index. If all the items are reliable and measure the same thing then the alpha factor is close to 1 (e.g., >0.70). The correlations between the items are given in the form of the Pearson correlation coefficient. Statistical analyzes were performed with the use of the STATISTICA 13.3PL TIBCO computer program.

### 2.6. Translation Process

Before starting the study in Poland, it was a translation of the scale into Polish conditions. The authors were given permission to translate the scale from the original author of the scale.

#### 2.6.1. Forward Translation

After the initial division of tasks, two nurses (HKS, and SK) prepared the material and submitted it for professional translation (medical language translator). Two separate translations (independent of each other) were made to check the correctness of the translated scale. The finalized version was proofread by two of the translators.

#### 2.6.2. Reconciliation

The translations were then merged in one preliminary version. The Polish version was adapted to a clinical setting without changing the meaning.

#### 2.6.3. Back Translation

The preliminary version was then back translated into English by an experienced and certified language teacher without knowledge to the original English version.

#### 2.6.4. Back Translation Review

The back translation of the preliminary Polish version was then thoroughly com-pared with the original text in regarding to the necessity of performing adjustments. This back translation showed no substantial deviations from the original after close comparison and assessment performer by the translating authors. The translation was carried out by an independent translator who accepted the version sent by the research team.

## 3. Results

### 3.1. Participants

In total, 450 questionnaires were distributed: 380 for nurses and 70 for doctors. The return of completed questionnaires was 65% (248) of nursing questionnaires and 67% (47) of doctors’ questionnaires. The questionnaires that were rejected due to incomplete and resignation from participation in the study amounted to 6%. Ultimately, 225 correctly completed nursing and 47 medical questionnaires qualified for the study. Information with data on the studied group is presented in Table 1.

### 3.2. Professional Autonomy in the Study Group

**Figure 1:**
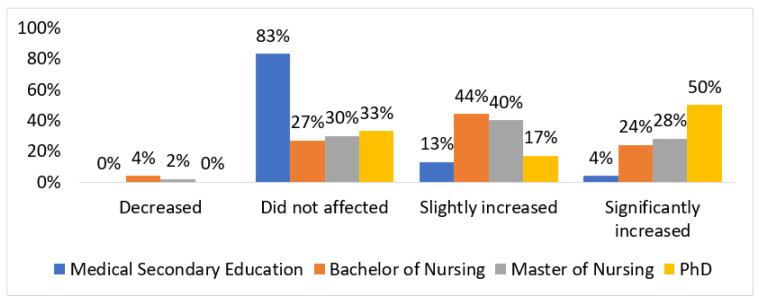
The impact of the COVID-19 pandemic on the level of professional independence depending on education.

In the study group, both nurses and doctors assessed the professional autonomy of nurses in the workplace high or very high, at 61% (*n* = 139) of nurses and 53% (*n* = 25) of doctors, respectively. A comparable percentage of both professional groups assessed the level of autonomy as average, at 36% (*n* = 118) of nurses and 38% (*n* = 18) of doctors, respectively. The low level of nurses’ autonomy was indicated by 9% (*n* = 4) of doctors and 2% (*n* = 4) of nurses. In this group, men significantly more often made such an assessment (12%) than women (1%). In the group of people who assessed the level of professional independence of nurses in the workplace as high, women were assessed significantly more often (52%) made such an assessment than men (35%). The positive impact of the pandemic on the level of professional independence of nurses was demonstrated for a comparable percentage of ICU doctors and nurses, 62% (*n* = 29) and 64% (*n* = 144), respectively. The analysis showed that respondents with lower medical education significantly more often did not notice the impact of the pandemic on professional independence (83%). The impact of the COVID-19 pandemic on the level of professional independence depending on education is presented in Figure 1.

When asked the question “How do you evaluate the level of your professional independence in the workplace?” 49% of respondents assessed the level of professional independence as high, including 52% of nurses and significantly less—34% of doctors. The group of 37% of the respondents assessed the level of professional independence as average and similar assessments were made in the group of 36% of nurses and 38% of doctors. The level of independence was assessed very highly by 11%, including 19% of respondents from the group of doctors and 9% of respondents from the group of nurses. 3% of the respondents assessed the level of independence as low. Doctors significantly more often assessed the level of professional independence as low than nurses (Table 2).

The distribution of assessments of professional independence in the workplace in the education groups was similar.

To the question “How has the COVID-19 pandemic affected the level of your professional independence?” the group of 37% of respondents answered that it has risen slightly (the same was true in the group of nurses and doctors). The 34% group claimed that the pandemic did not affect the level of professional independence (it was similar in the group of nurses and doctors). The group of 26% claimed that the pandemic had influenced the level of professional independence in such a way that it had increased significantly (and it was similar in the group of nurses and doctors). Only a group of 3% of respondents claimed that the pandemic reduced the level of professional independence (the same was true for nurses and doctors). The results are presented in Table 3.

When asked, “How much has the COVID-19 pandemic affected the level of trust in your nurse–nurse relationship in your team,” 47% of respondents assessed that confidence had decreased, and 28% said that trust had improved slightly. A group of 20% of respondents said that trust had improved a lot, and 4% claimed that trust had not changed (Table 4).

On the basis of the conducted research, it could be concluded that the respondents with the highest average age and the biggest work experience, both in the profession and ITU, has not noticed the impact of the pandemic on the level of professional independence. On the other hand, respondents at a younger age and less seniority claimed that the pandemic had contributed to an increase in professional independence in the workplace. The results are presented in Table 5.

Doctors less frequently than nurses assessed the level of professional independence of nurses as high (34%, *n* = 16); however, they assessed nurses’ autonomy at a very high level twice as often as nurses themselves (19%, *n* = 9). In the assessment of trust in the nursing team, 49% of the respondents (*n* = 112) indicated an increase in the level of trust, while 47% of the respondents (*n* = 104) indicated that the level of trust in the ICU team decreased.

The impact of the COVID-19 pandemic on the level of professional independence of the respondents in terms of gender was also assessed. Both women and men noticed a slight increase in professional independence during the pandemic. The results are presented in Table 6.

### 3.3. Validation Values of the DPBS Scale

Data was collected for the DPBS scale tests. Reliability analysis was performed in order to assess the reliability of the analyzed scale, calculating the Cronbach’s alpha index. If all the items are reliable and measure the same thing then the alpha factor is close to 1 (e.g., >0.70). The correlations between the items are given as Pearson’s correlation index r. Statistical analyzes were performed with the use of the STATISTICA 13.3PL TIBCO computer program. The results of the tool validation are presented in Table 7 and Table 8.

The values of skewness and kurtosis are in the range (−1.5–1.5), which allows us to treat the scale distribution and subscales as a normal distribution and allows the use of parametric tests and methods. The description is presented in Table 9.

The DPBS subscales are strongly, positively correlated with DPBS at a statistically significant level (Table 10)

The DPBS subscales are significantly correlated with each other. In a strong, significant and positive correlation there are all subscales of independence (r = 0.536), self-actualization (r = 0.713) and self-esteem (r = 0.587) with the readiness scale. The self-correlation scale (r = 0.405) is significant in the average relationship with self-reliance, and the self-esteem scale (r = 0.487) is in the average correlation relationship with self-actualization (Table 11).

## 4. Discussion

Work autonomy is a very important element and the basis of Evidence Based Practice, especially in medical professions. Our research proves that the COVID-19 pandemic has contributed to an increase in autonomy, especially among young people. Research by Goolsby et al. confirms the credibility of the Dempster Practice Behavior Scale and at the same time proves that the use of the scale brings benefits related to the study of work autonomy [9]. In their review of the literature, Choi et al. emphasize that autonomy among nurses is closely related to the work dynamics and patient care. The authors emphasize that it is necessary to conduct further research on autonomy and its dimensions in order to determine the direction of nurses’ work in the future [10]. In their work, Cajulis et al. also bring into light the topic of autonomy. According to the results, 41% of the respondents showed a very high level of autonomy and 19% had a moderate level of autonomy. Demographic data in the study by Cajulis et al. had no statistically significant correlation with the results of the autonomy level [11]. In the study group, both nurses and doctors assessed the professional autonomy of nurses in the workplace as high or very high, at 61% (*n* = 139) of nurses and 53% (*n* = 25) of doctors, respectively. In the latest publication by Gharaaghaji Asl et al., after examining nurses in a nursing school in Iran, moderate autonomy and stress at work were shown. Occupational autonomy showed a significant positive correlation with occupational stress and a significant positive correlation with work experience at the ICU [12]. In a study by Aghamohammadi et al. the autonomy of nurses working in ICU was at a moderate level [7]. In turn, in the study by Shohani et al. it was shown that the professional autonomy of ICU nurses was high [13]. In the authors’ own study, the respondents with the highest seniority, both in their profession and in the ICU experience, did not notice the impact of the pandemic on the level of professional independence. Motamed-Jahromi et al. in their publication describe that nurses assessed their professional autonomy relatively well [14]. In the United States, nurses have been shown to have high professional autonomy compared with Cypriot nurses [15,16]. Many of the issues related to differences in autonomy in different countries may result from the culture, curriculum or procedures that prevail in individual countries. During the COVID-19 pandemic, many medical professions counted on an increase in respect and remuneration due to the difficult period, which in fact the time of the pandemic was. Thanks to this difficult period, it can be noticed that the work performed by specialists working with COVID-plus patients was appreciated in the media and in publications due to the effort and heroic struggle for the sick [17]. Nurses and doctors face many professional difficulties due to the pandemic. This time also influenced the physician-centered health model, and nurses continued to be secondary heroes of the pandemic. Such a situation may lead to a weakened motivation and an increase in dissatisfaction in this group of nurses [18]. Despite the fact that nurses highly rated their autonomy in this study, it should be remembered that officially, nurses will never achieve full working autonomy compared with clinicians [19,20,21]. In addition to autonomy, the authors’ study examined nurses in terms of trust in this group. Almost half of the nurses replied that confidence had decreased during the pandemic, and 28% of respondents said that trust had improved slightly. Despite the results of their own research, other authors emphasize that the public’s trust towards health care workers has significantly improved [22]. It should be emphasized that trust is the most important element of cooperation in every team, and work in critical situations increases trust among colleagues [23,24].

In future studies, it should be assessed whether the increase in the level of autonomy among nurses was caused by the COVID-19 pandemic and related staff shortages, or it is a result of an increase in the level of education and qualifications among nurses, and whether this trend will continue to the benefit of patients. This is an important issue for policymakers, who should define the level of autonomy and note that the autonomy of nurses is very beneficial for both patients and the entire therapeutic team.

## 5. Conclusions

The results of the research show that the DPBS tool has the right validation parameters that allow the use of this scale to assess the autonomy of work among Polish nurses. Nursing autonomy has increased during the pandemic. This research showed that autonomy in group of nurses was higher during the COVID-19 pandemic in the opinion of nurses, but not in all groups. The nurses’ autonomy was assessed significantly lower by the doctors working with them. This tool DPBS can be used to conduct further research on professional autonomy in other areas of nursing.

## 6. Limitations

Because the survey’s findings were limited to some ICUs in Poland, the results may only be applied to specific institutions and may not accurately reflect nurses’ views of the country’s health services. Further research could be carried out in different size hospitals, provincial hospitals, or private hospitals to see if there are differences. In addition, future qualitative research that will thoroughly examine the autonomy of nurses would benefit in more accurate results.

## Figures and Tables

**Table 1:** Group characteristics.

Factor	Nurses		Doctors	
Gender	*n* = 225	%	*n* = 47	%
Woman	204	91	25	53
Man	21	9	22	47
Education				
Medical secondary	24	11	-	
Bachelor of Nursing	70	31	-	
Master of Nursing/Medical Doctor	130	58	42	89
Doctorate	1	0,4	5	11
Qualifications				
Completed anesthetic nursing course	104	46	-	
During the anesthetic nursing course	12	5	-	
Completed specialization in anesthetic nursing/anaesthesiology	102	45	27	57
In the course of specialization in anesthetic nursing/anaesthesiology	40	18	20	43
Work experience in the profession				
1–5 years	67	30	12	26
6–10 years	49	22	13	28
11–15 years	30	13	8	17
16–20 years	18	8	5	11
21–25 years	22	10	2	4
26–30 years	22	10	4	8
31 years and more	17	8	3	6
Work experience in the ICU				
1–5 years	83	37	26	55
6–10 years	48	21	6	13
11–15 years	33	15	5	11
16–20 years	25	11	3	6
21–25 years	16	7	1	2
26–30 years	12	5	5	11
31 years and more	8	4	1	2

**Table 2:** Characteristics of the assessment of professional independence of nurses in the workplace in the group of respondents.

How Do You Assess the Level of Your (Nurses) Professional Independence in the Workplace?	Nurses		Doctors		Overall	
	*n*	%	*n*	%	*n*	%
low	4	2%	4	9%	8	3%
average	82	36%	18	38%	100	37%
high	118	52%	16	34%	134	49%
very high	21	9%	9	19%	30	11%
Overall	225	100%	47	100%	272	100%
χ^2^ = 10.35, *p* < 0.01						

**Table 3:** Characteristics of the assessment of professional independence in the workplace in the education group.

How Do You Assess the Level of Your (Nurses) Professional Independence in the Workplace?	Medium		Bachelor Degree		Master		Doctorate	
	*n*	%	*n*	%	*n*	%	*n*	%
low	0	0%	1	1%	6	3%	1	17%
average	6	25%	32	46%	60	35%	2	33%
high	17	71%	31	44%	84	49%	2	33%
very high	1	4%	6	9%	22	13%	1	17%
Overall	24	100%	70	100%	172	100%	6	100%
χ^2^ = 11.77, *p* < 0.22.								

**Table 4:** Characteristics of the assessment of the level of trust in the team in the nurse–nurse relationship in the group of respondents.

How Has the COVID-19 Pandemic Affected the Level of Trust in Your Nurse–Nurse Relationship Team?	Nurses	
	*n*	%
Confidence has not changed	9	4%
Confidence has dropped	104	47%
Confidence has improved slightly	66	29%
Confidence has improved a lot	46	20%
Overall	225	100%

**Table 5:** The impact of the COVID-19 pandemic on the level of professional independence in particular age and work experience groups.

How Has the COVID-19 Pandemic Affected Your Professional Independence?	Age		Work Experience in the Profession		Work Experience in the ICU	
	x	SD	x	SD	x	SD
It has lowered	38.0	8.7	15.1	11.3	12.6	10.7
It hasn’t	41.8	9.7	18.4	10.7	15.2	9.9
Is has risen slightly	34.7	8.6	10.0	8.5	8.1	7.8
It has risen greatly	35.2	9.7	11.7	9.8	8.6	7.9

**Table 6:** The impact of the COVID-19 pandemic on independence in the group of women and men.

How Has the COVID-19 Pandemic Affected Your Professional Independence?	Women		Man		Overall	
	*n*	%	*n*	%	*n*	%
It has decreased	5	2%	2	5%	7	3%
It has had no effect	78	34%	14	33%	92	34%
It has risen slightly	84	37%	19	44%	103	38%
It has risen greatly	62	27%	8	19%	70	26%
Overall	229	100%	43	100%	272	100%

**Table 7:** Cronbach’s Alpha coefficient for the DPBS scale.

DPBS	Number of Items on the Scale	Number of Cases	Results of Cronbach Alfa	Average Correlation between Items	Mean	Standard Deviation
	30	225	0.904	0.24	117.17	14.12

**Table 8:** Cronbach’s Alpha coefficient in the individual subscales.

DPBS	Item Total Correlation	Item Total Correlation Alfa Cronbach’s Alpha after Item Removal
Readiness—Items-11 Cronbach’s alpha = 0.864		
2	0.524	0.853
4	0.536	0.852
6	0.561	0.851
7	0.600	0.848
11	0.531	0.853
12	0.495	0.855
20	0.632	0.846
21	0.666	0.843
22	0.537	0.853
27	0.518	0.854
29	0.546	0.852
Independence—Items–7 Cronbach’s alpha = 0.614		
8	0.423	0.543
13	0.427	0.543
15	0.244	0.604
17	0.393	0.554
24	0.247	0.601
26	0.229	0.610
28	0.331	0.577
Self-Realization—Items–9 Cronbach’s alpha = 0.795		
1	0.372	0.775
3	0.522	0.757
9	0.540	0.752
10	0.552	0.750
14	0.554	0.750
16	0.531	0.753
18	0.249	0.806
25	0.567	0.749
30	0.444	0.766
Self-esteem—Items–3 Cronbach’s alpha = 0.515		
5	0.221	0.204
19	0.165	0.664
23	0.336	0.100

**Table 9:** Subscale measures.

DPBS	M	Me	SD	Min	Max	Skewness	Kurtosis
Readiness	42.18	43	6.19	26	55	−0.25	0.27
Independence	24.84	25	4.03	14	35	−0.02	0.27
Self Realization	38.50	39	4.11	20	45	−0.63	0.27
Self-esteem	11.50	12	2.31	3	15	−0.70	0.27
DPBS	117.02	118	13.53	71	150	−0.33	0.27

**Table 10:** Pearson’s r correlation subscales with DPBS.

Pearson’s r Correlation with DPBS	r	*p*
Readiness	0.934	0.0001
Independence	0.700	0.0001
Self Realization	0.834	0.0001
Self-esteem	0.647	0.0001

**Table 11:** Pearson’s r correlation between subscales.

Pearson’s r Correlation		Readiness	Independence	Self Realization
Readiness Independence	r			
	*p*			
Self Realization	r	0.536		
	*p*	0.0001		
Readiness Independence	r	0.713	0.405	
	*p*	0.0001	0.0001	
Self Realization	r	0.587	0.198	0.487
		0.0001	0.0001	0.0001

## Data Availability

The authors declare that the data from this study are available from the author H.K-S. upon request.
